# Supplementary material for: Targeted genome editing in vivo corrects a Dmd duplication restoring wild‐type dystrophin expression
Source: EMBO Mol Med. 2021 Mar 16;13(5):e13228. doi: 10.15252/emmm.202013228 (PMC8103086; doi:10.15252/emmm.202013228)
Supplement: Supplementary file 1 — Appendix [file EMMM-13-e13228-s001.pdf]

Table of content

Appendix Figure S1: Overview of the procedure utilized to generate the *Dup18-30i* mouse model.

Appendix Figure S2: The *Dup18-30i* mouse model shows abnormal splicing of the *Dmd* transcript which was corrected in the *Dup18-30* mouse model.

Appendix Figure S3: The *Dup18-30i* mouse model has a dystrophic phenotype and decreased muscle strength.

Appendix Figure S4: Amplicon PCR deep sequencing analysis for i21 sgRNA on-target activity.

Appendix Table S1: Structural variants (SV) identified by WGS in the *Dup18-30i* mouse model.

Appendix Table S2: Structural variants (SV) identified by WGS in the *Dup18-30* mouse model.

Appendix Table S3: Top 8 sgRNAs screened in vitro in N2A cells ranked by activity.

Appendix Table S4: Summary of indels formation within the guide target site and at the top 11 off target sites.

Appendix Table S5: sgRNA sequences used for mouse model generation and duplication removal.

Appendix Table S6: Primers used in this study.

Appendix Table S7: Exact p-values table.

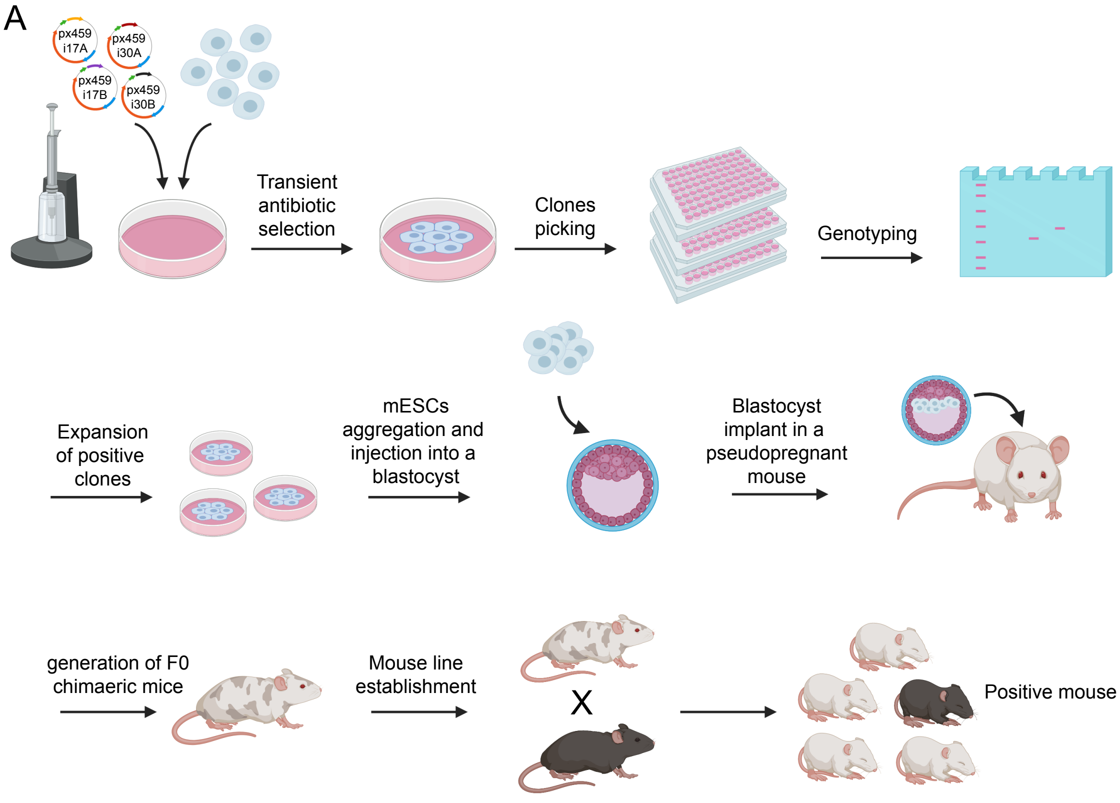

Appendix Figure S1. Overview of the procedure utilized to generate the *Dup18-30i* mouse model.

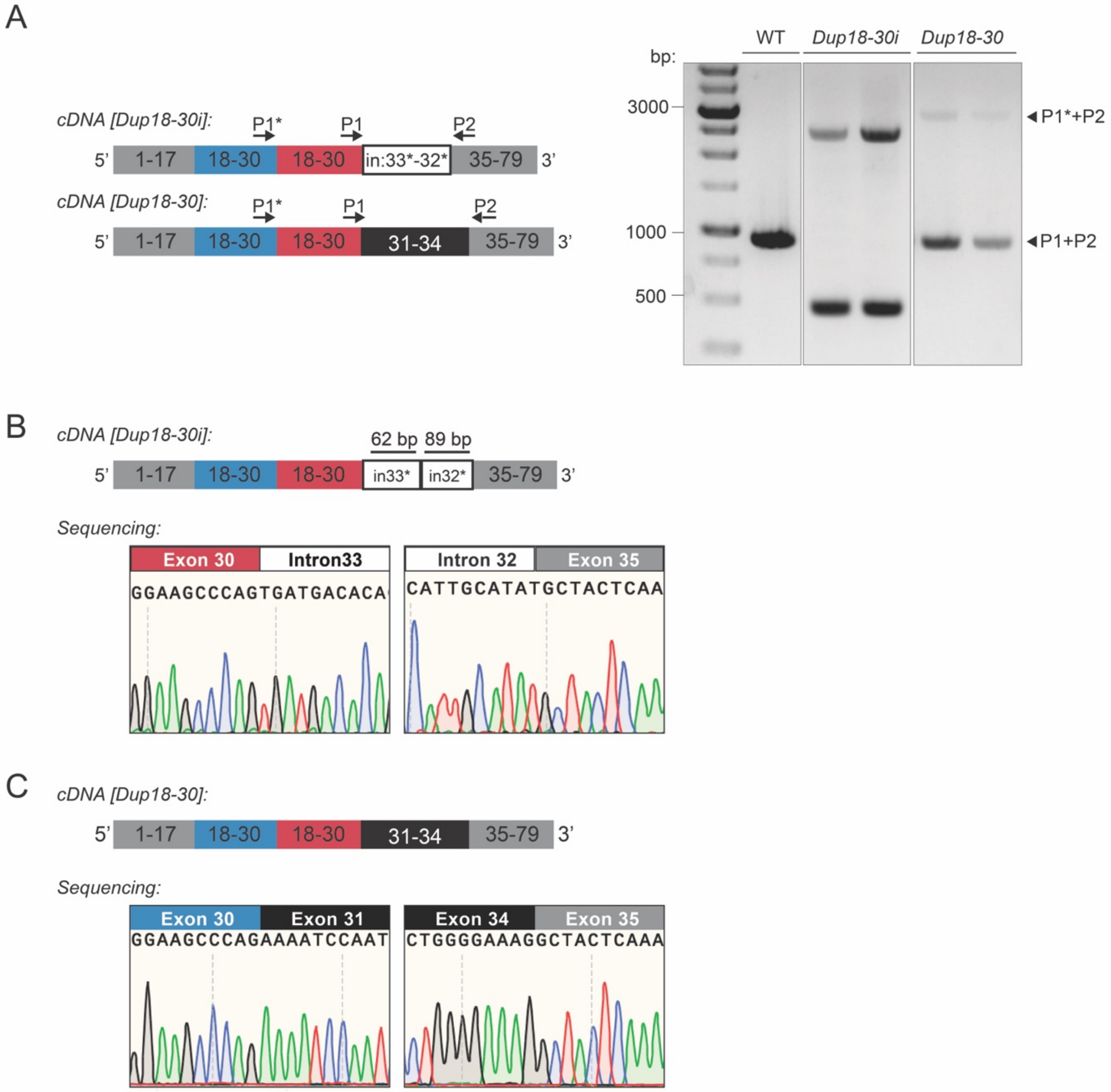

Appendix Figure S2. The *Dup18-30i* mouse model shows abnormal splicing of the *Dmd* transcript which was corrected in the *Dup18-30* mouse model. A) Schematic representation (not to scale) of the *Dmd* mRNA in *the Dup18-30i* and *Dup18-30* mouse models (left). RT-PCR on cDNA from TA muscle of WT, *Dup18-30i*, and *Dup18-30* mice (right). Arrows correspond to primers in exons 29 and 35. B) The inclusion of two pseudoexons derived from regions of intron 33 and intron 32 and the exclusion of the exons from 31 to 34 was confirmed via Sanger sequencing of the junction between exon 30 and intron 33 (left) and intron 32 and exon 35 (right). C) Verification of *Dmd* splicing in the *Dup18-30* mice via Sanger sequencing of the junction between exons 30 and 31 (left) and exons 34 and 35 (right).

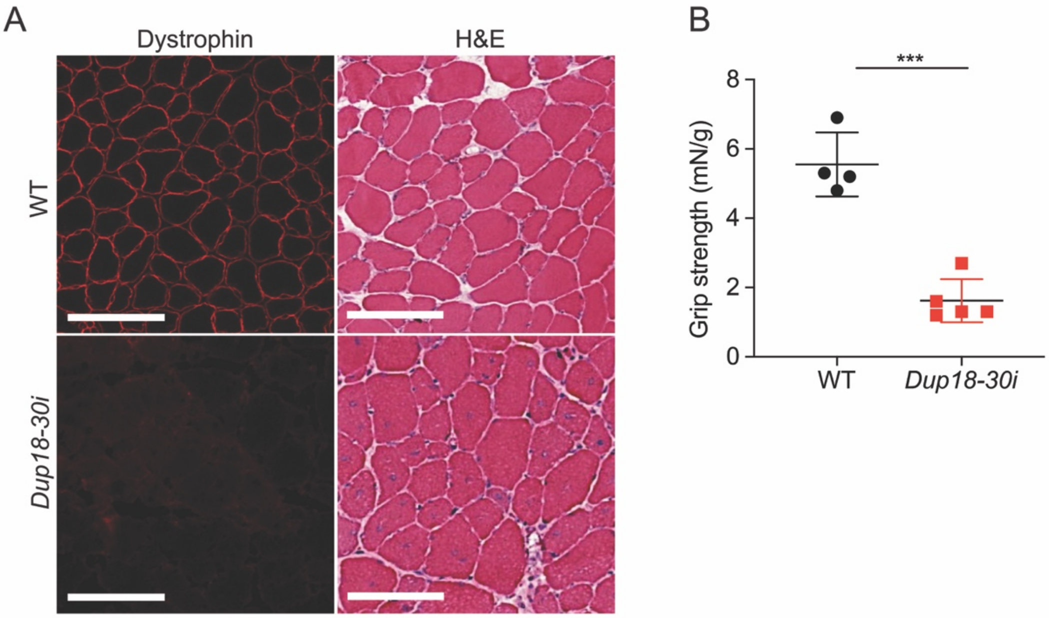

Appendix Figure S3. The *Dup18-30i* mouse model has a dystrophic phenotype and decreased muscle strength. A) TA muscle cross-sections were analyzed for localization of dystrophin and general muscle architecture by immunofluorescence (left) and H&E (right) staining, respectively. A representative sample of a 4-week-old WT and age matched *Dup18-30i* mouse is shown. Scale bars, 100 µm. B) Grip Strength analysis of 4-week-old WT and age matched *Dup18-30i*.

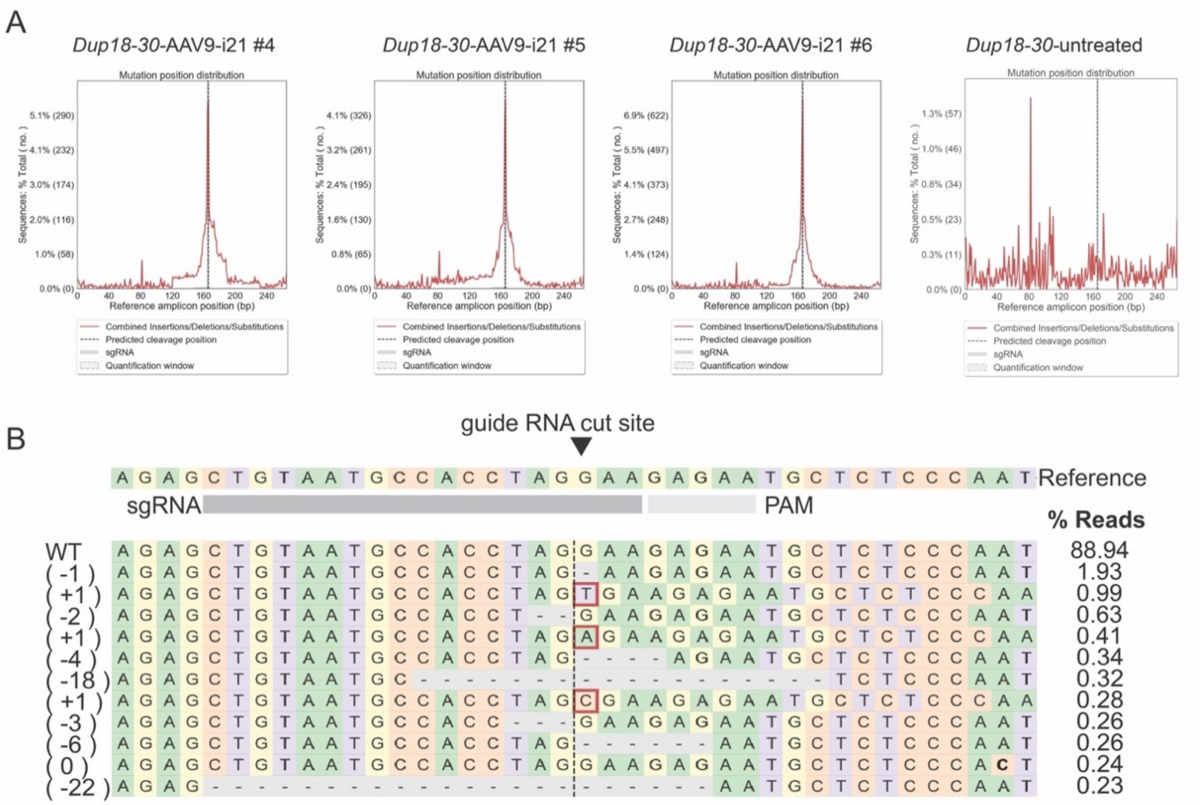

C

Tissue	Sample	% Indels	Average %Indels
TA	*Dup18-30*-AAV9-i21 #1	2.7	3.07 (±0.010)
	Dup18-30-AAV9-i21 #2	4.65	
	Dup18-30-AAV9-i21 #3	2.27	
	Dup18-30-AAV9-i21 #4	2.84	
	Dup18-30-AAV9-i21 #5	3.99	
	Dup18-30-AAV9-i21 #6	1.97	
	Dup18-30-untreated #1	0.29	0.29
Heart	*Dup18-30*-AAV9-i21 #1	4.66	5.67 (±0.018)
	Dup18-30-AAV9-i21 #2	5.06	
	Dup18-30-AAV9-i21 #3	3.79	
	Dup18-30-AAV9-i21 #4	5.22	
	Dup18-30-AAV9-i21 #5	6.5	
	Dup18-30-AAV9-i21 #6	8.8	
	Dup18-30-untreated #1	0.44	0.44

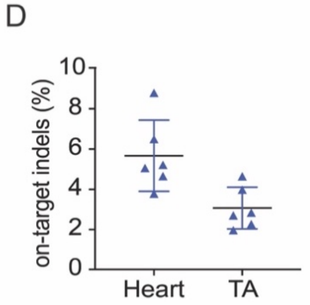

Appendix Figure S4. Amplicon PCR deep sequencing analysis for i21 sgRNA on-target activity. A) Representative graphs generated by the CRISPResso2 software analysis of i21 sgRNA on-target site activity in the heart of *Dup18-30* mice. On the x-axis is represented the indels distribution across the PCR amplicons, where dotted lines represent predicted Cas9 cleavage sites in treated (AAV9-i21 #4, #5, #6) and untreated *Dup18-30* mice. B) Representative deep sequencing analysis of the PCR amplicons generated across the i21 sgRNA target site in the heart of a *Dup18-30* Cas9+i21 treated mouse. C) Analysis of indels formation at the i21 sgRNA target site. Sequencing of an untreated *Dup18-30* mouse was used to determine the background of the sequence analysis. D) Plot summarizing the detected indels events in TA and heart. *Dup18-30* treated mice, n = 6.

Appendix Table S1. Structural variants (SV) identified by WGS in the *Dup18-30i* mouse model.

Chr	Pos start	Pos end	SV length	SV type	Loci Annotation
X	83737128	83738567	-1439	DEL	Intron 17 del
X	83737872	83874709	136837	DUP	Dup 18-30
X	83874712	83876784	-2703	DEL	Intron 30 del
X	83737876	83737966	91	INS	Insertion of part of intron 17
X	83737966	83737999	33	INS	Insertion of 33 bp
X	83876785	83888834	12049	INV	Inv intron 30 to intron 34

Appendix Table S2. Structural variants (SV) identified by WGS in the *Dup18-30* mouse model.

Chr	Pos start	Pos end	SV length	SV type	Loci Annotation
X	83737128	83738567	-1439	DEL	Intron 17 del
X	83737872	83874709	136837	DUP	Dup 18-30
X	83874712	83876784	-2703	DEL	Intron 30 del
X	83737876	83737966	91	INS	Insertion of part of intron 17
X	83737966	83737999	33	INS	Insertion of 33 bp
X	83888848	83895366	6519	INV	Inversion part of intron 34
X	83895367	83895465	-118	DEL	Intron 34 del
X	83895466	83896623	1158	INV	Intron 34 inversion
X	83896624	83898824	-2079	DEL	Intron 34 del

Table S3. Top 8 sgRNAs screened in vitro in N2A cells ranked by activity.

Guide name	Guide sequence	ICE score
mDmd i21-1 (i21)	GCTGTAATGCCACCTAGGAA	17%
mDmd i21-2	AAGTACACTTAAATGCCGGT	9.5%
mDmd i29-1	GGAGACTTGCCCCAATGTAG	7%
mDmd i29-2	GTAGGTAGAGAGTCCTACAT	1%
mDmd i27-1	TGGAGCATAAGTAACCTCAC	N/A
mDmd i29-3	CTAGGTCGTGTAGACATCGC	N/A
mDmd i21-3	AAGAGGATCGCGACTACTAG	N/A
mDmd i20-1	GATGGCCTTAAGGCCCCACT	N/A

Appendix Table S4. Summary of indels formation within the guide target site and at the top 11 off target sites. All the OT4 samples and OT5 ctrl did not meet the filtering criteria.

**Target**	**sgRNA sequence**	**PAM**	**Gene**	**Chr**	**#MM**	**% Indels**	**Treated/ Untreated**
TA - ON	GCTGTAATGCCACCTAGGAA	GAGAA	Dmd – in21	X	0	3.07 (±0.010)	10.59
H - ON	GCTGTAATGCCACCTAGGAA	GAGAA	Dmd – in21	X	2	5.67 (±0.018)	12.89
OT1	GCAGTAATGCCACCAAGGAA	AAGGA	None	7	3	0.61 (±0.001)	1.12
OT2	TCTGAAATGCCACCTAGAAA	ATGGG	None	10	3	0.23 (±0.000)	0.76
OT3	CCTGTAATGCCAACTTGGAA	AGGAA	None	7	3	0.09 (±0.000)	1.33
OT4	GCTGAAATGCCCCTTAGGAA	TAGGA	None	7	3	N/A	N/A
OT5	GCAGGAATGGCACCTAGGAA	GGGGA	None	1	3	0.33 (±0.000)	N/A
OT6	GCTGTAGAGCCACGTAGGAA	ATGGA	None	15	3	0.08 (±0.000)	0.70
OT7	GCTGTAATGCCAGTTAAGAA	ATGGA	None	11	3	0.24 (±0.000)	1.28
OT8	ACTGTCATGCCAACTAGGAC	TAGAA	None	7	4	0.25 (±0.000)	1.15
OT9	AATGTAATCCCACCTAGGAC	ATGGG	None	10	4	0.32 (±0.000)	1.20
OT10	TCTGAAATGCCACGTAGGCA	TGGAG	None	10	4	0.10 (±0.000)	1
OT11	TCTGTAATGTCACCAAGGAG	ACGAG	ENSMUSG00000076864	14	4	0.13 (±0.000)	1.06

Appendix Table S5. sgRNA sequences used for mouse model generation and duplication removal.

**Guide name**	**Oligo sequence**	**PAM**	**Purpose**
mDmd in17 g A	GCATGGCGCAAAGGTCAAGA	AGG	Mouse model generation
mDmd in17 g B	AATACTACTAGCTCACCATC	TGG	
mDmd in30 g A	ACTGGTGAAATCGTGCCCGG	AGG	
mDmd in30 g B	GTCCTAAATTTGCAGAACGA	TGG	
mDmd inv g A	CTACTAGCTGAATCAAAAGA	TGG	
mDmd inv g B	AAAGAATCGACCCAAGCCTC	TGG	
mDmd i21	GCTGTAATGCCACCTAGGAA	GAGAAT	Duplication correction

Appendix Table S6. Primers used in this study.

Primer name	Primer sequence	Purpose
mDmd in30 junct F	GCCTGAGAAGCATCATACCACAACG	amplification of gDNA duplication junction
mDmd in17 junct R	GAGCATGAAACGAAGCCAGAGATTAGAC	
mDmd in30 seq F	CCTCGGACTCAAGGTCTTTCGAAG	
mDmd ex30 F	GAGTATCCAGTCTGCCCAGGAAATTG	amplification of cDNA duplication junction
mDmd ex18 R	CTTGCAAGTCTGAGATGTTGCCTTC	
mDmd ex30 F	GAGTATCCAGTCTGCCCAGGAAATTG	amplification of cDNA duplication exon 30-30
mDmd ex30 R	ATTTGAGCTGCATCCACCTTGTCAG	
mDmd ex16 F	GATCTACTTTCGGCACTGAAAAAT	amplification of cDNA duplication exon 16-17
mDmd ex17 R	GTTTTACCATGATTTGTTCCCTTG	
mDmd ex29 F	ACTGATCAATGAGGAGCTTGAGACG	amplification of cDNA duplication exon 29-35
mDmd ex35 R	CTTCTACCAAGGTTTCTTTCTTGC	
mDmd i21-1 ICE F	ATTTTATTTTTAAGGTGGGCTGCT	ICE analysis
mDmd i21-1 ICE R	TAAGTACACTTAAATGCCGGTCTG	
mDmd i21-2 ICE F	CCTTTCACCACCAGAAGCTACAC	
mDmd i21-2 ICE R	CATTGGTCAGTTTTGTGAAGCAC	
mDmd i21-3 ICE F	GGGATACATGTGCACTAGATTAGCC	
mDmd i21-3 ICE R	AATTCATTTTGGAAAAGATAGGCAAC	
mDmd i29-1 ICE F	GCACCTGGACTTTTGGGGGTATTTC	
mDmd i29-1 ICE R	GGCACTTGCAGATGTCAAGAAG	
mDmd i29-2 ICE F	GGTGCTGGCTTACAGTTGAAAGC	
mDmd i29-2 ICE R	GGAAGACTATGAGGAACATTGCCTG	
mDmd i29-3 ICE F	TATGGAGCCTACCCTTGAGTCTG	
mDmd i29-3 ICE R	ACTTTTGCTTGACTCTGCTCAGG	
mDmd i27-1 ICE F	TCTCCCCCATTTCTTCTTGTATCTCCAC	
mDmd i27-1 ICE R	TGATCAAGGTATAGACAACAAAAGGGGC	
mDmd i20-1 ICE F	CAGGCTTATCAACAGCAATACCC	
mDmd i20-1 ICE R	GCTTAGCGGAAATCAGACTAAAATG	
mDmd in30 qPCR F	TCTGTTTCTGGCATACTGTCTTGT	qPCR DNA editing
mDmd in17 qPCR R	TAAGCAATGAACTCCAAGAAATGA	
mDmd ex16 qPCR F	GATCTACTTTCGGCACTGAAAAAT	
mDmd ex16 qPCR R	TGCTGAACTCTTTTCAAGTTTTTG	
mDmd ex33-34 R	TGTTACCTTCGCACCCAACTCATTG	RT-PCR RNA editing
mDmd ex16 F	AGCCAACCATGGAAAAACTAAGTTCAC	
mDMD ex30 qPCR F	TTATATCACTGACAAGGTGGATGC	qRT-PCR RNA editing
mDMD ex18 qPCR R	TACAGCTTCTGAACGAGTAATCCA	
mDmd ex16 qPCR F	GATCTACTTTCGGCACTGAAAAAT	
mDmd ex16-17 qPCR R	CTGTGAAATTTGTGCTGAACTCTT	

mDmd i21 OT1 F	TCGTCGGCAGCGTCAGATGTGTATAAGAGACAGCTAACAGTCTCGATTTTCCCACA	Deep sequencing
mDmd i21 OT1 R	TCGTCGGCAGCGTCAGATGTGTATAAGAGACAGCTAACAGTCTCGATTTTCCCACA	
mDmd i21 OT2 F	TCGTCGGCAGCGTCAGATGTGTATAAGAGACAGAGTCATGAGAGGCATCGTGAATA	
mDmd i21 OT2 R	GTCTCGTGGGCTCGGAGATGTGTATAAGAGACAGCCCTGCCTACAAAAACGAAAAC	
mDmd i21 OT3 F	TCGTCGGCAGCGTCAGATGTGTATAAGAGACAGGCTCATCTGGTCTCCGTTTTATT	
mDmd i21 OT3 R	GTCTCGTGGGCTCGGAGATGTGTATAAGAGACAGCCAACCAGTTTTACTGTGTCGTA	
mDmd i21 OT4 F	TCGTCGGCAGCGTCAGATGTGTATAAGAGACAGGGAAACTATACAGCTATGGGGATG	
mDmd i21 OT4 R	GTCTCGTGGGCTCGGAGATGTGTATAAGAGACAGGTGTGTGTATGTGCTGAAATGC	
mDmd i21 OT5 F	TCGTCGGCAGCGTCAGATGTGTATAAGAGACAGGTTAGAGGGCTACCAACAAAGTG	
mDmd i21 OT5 R	GTCTCGTGGGCTCGGAGATGTGTATAAGAGACAGTGATATTTCCTTTCTGTGGCTTT	
mDmd i21 OT6 F	TCGTCGGCAGCGTCAGATGTGTATAAGAGACAGGCACAAACACTCTCACAGACATAA	
mDmd i21 OT6 R	GTCTCGTGGGCTCGGAGATGTGTATAAGAGACAGATGTATGCCTGCATATCACTTGC	
mDmd i21 OT7 F	TCGTCGGCAGCGTCAGATGTGTATAAGAGACAGCATTATCACAAGGCTGGATGTTT	
mDmd i21 OT7 R	GTCTCGTGGGCTCGGAGATGTGTATAAGAGACAGGGTGGGAAGATACACTCCTTACC	
mDmd i21 OT8 F	TCGTCGGCAGCGTCAGATGTGTATAAGAGACAGCAGTGTGCTCTAGGCAATACCTC	
mDmd i21 OT8 R	GTCTCGTGGGCTCGGAGATGTGTATAAGAGACAGGAGTGCGGTACTGGTAACAGAGT	
mDmd i21 OT9 F	TCGTCGGCAGCGTCAGATGTGTATAAGAGACAGGTAAAACAGCGGTCTTTCAGCTA	
mDmd i21 OT9 R	GTCTCGTGGGCTCGGAGATGTGTATAAGAGACAGTAGCCTGAGCTTTTCAATGAGAC	
mDmd i21 OT10 F	TCGTCGGCAGCGTCAGATGTGTATAAGAGACAGTGCAAGGTTCTCTGTGAGTAATG	
mDmd i21 OT10 R	GTCTCGTGGGCTCGGAGATGTGTATAAGAGACAGACACCCTACAAGCTTCATCAAAG	
mDmd i21 OT11 F	TCGTCGGCAGCGTCAGATGTGTATAAGAGACAGAGAAGGAGATGAAGTCACCATGA	

mDmd i21 OT11 R	GTCTCGTGGGCTCGGAGATGTGTATAAGAGACAGAGGGAACTTTGTTGCATTTTCTT	
mDmd i21 ON-target F	TCGTCGGCAGCGTCAGATGTGTATAAGAGACAGTCCACATGTAACTGAGGCATCC	
mDmd i21 ON-target R	GTCTCGTGGGCTCGGAGATGTGTATAAGAGACAGGAGCTGAGTGGAGCAGGACT	

Appendix Table S7. Exact p-values table.

Figure	Sample	Comparison	p-value
2C	TA	WT vs *Dup18-30*	5.7598E-17
	Triceps	WT vs *Dup18-30*	1.7553E-15
2D	TA	WT vs *Dup18-30*	1.0023E-07
	Triceps	WT vs *Dup18-30*	3.4552E-07
2E		WT vs *Dup18-30*	0.0004
2F		WT vs *Dup18-30*	0.0002
2G		WT vs *Dup18-30*	0.0002
2H		WT vs *Dup18-30*	0.0002
3C	Heart	*Dup18-30* untreated vs *Dup18-30* Cas9+i21	0.0094
	TA	*Dup18-30* untreated vs *Dup18-30* Cas9+i21	0.1536
	Triceps	*Dup18-30* untreated vs *Dup18-30* Cas9+i21	0.0853
3D	Heart	*Dup18-30* untreated vs *Dup18-30* Cas9+i21	0.0005
	TA	*Dup18-30* untreated vs *Dup18-30* Cas9+i21	0.2827
	Triceps	*Dup18-30* untreated vs *Dup18-30* Cas9+i21	0.2375
3I	Heart	*Dup18-30* untreated vs *Dup18-30* Cas9+i21	0.0483
	TA	*Dup18-30* untreated vs *Dup18-30* Cas9+i21	0.0433
3J	Heart	*Dup18-30* untreated vs *Dup18-30* Cas9+i21	3.8348E-10
	TA	*Dup18-30* untreated vs *Dup18-30* Cas9+i21	1.2711E-05
4C	TA	*Dup18-30* untreated vs *Dup18-30* Cas9+i21	5.0530E-06
	Triceps	*Dup18-30* untreated vs *Dup18-30* Cas9+i21	0.0004
	Diaphragm	*Dup18-30* untreated vs *Dup18-30* Cas9+i21	1.8753E-05
5B		WT vs *Dup18-30* untreated	2.0555E-05
		Dup18-30 untreated vs *Dup18-30* Cas9+i21	0.0002
		WT vs *Dup18-30* Cas9+i21	0.0042
5C		WT vs *Dup18-30* untreated	4.1478E-08

		Dup18-30 untreated vs *Dup18-30* Cas9+i21	0.0027
		WT vs *Dup18-30* Cas9+i21	6.0724E-08
5D		WT vs *Dup18-30* untreated	0.0058
		Dup18-30 untreated vs *Dup18-30* Cas9+i21	0.0016
		WT vs *Dup18-30* Cas9+i21	0.9776
5E		WT vs *Dup18-30* untreated	8.1874E-06
		Dup18-30 untreated vs *Dup18-30* Cas9+i21	2.2443E-05
		WT vs *Dup18-30* Cas9+i21	0.1113
5F		WT vs *Dup18-30* untreated	1.5023E-05
		Dup18-30 untreated vs *Dup18-30* Cas9+i21	6.6218E-05
		WT vs *Dup18-30* Cas9+i21	0.0722
EV1C		WT vs *Dup18-30*	0.0031
EV1D		WT vs *Dup18-30*	0.0002
EV2C	Triceps	*Dup18-30* untreated vs *Dup18-30* Cas9+i21	0.0436
	Diaphragm	*Dup18-30* untreated vs *Dup18-30* Cas9+i21	0.0172
EV2E	Triceps	*Dup18-30* untreated vs *Dup18-30* Cas9+i21	8.6808E-05
	Diaphragm	*Dup18-30* untreated vs *Dup18-30* Cas9+i21	9.3797E-06
EV4B		*Dup18-30* untreated vs *Dup18-30* Cas9+i21	2.4572E-05
EV5A		WT vs *Dup18-30* untreated	8.0388E-07
		Dup18-30 untreated vs *Dup18-30* Cas9+i21	0.0002
		WT vs *Dup18-30* Cas9+i21	0.6436
EV5B		WT vs *Dup18-30* untreated	0.0013
		Dup18-30 untreated vs *Dup18-30* Cas9+i21	0.0391
		WT vs *Dup18-30* Cas9+i21	0.3023

Appendix S3B		WT vs *Dup18-30* untreated	0.0001
