# Supplementary material for: Targeted genome editing in vivo corrects a Dmd duplication restoring wild‐type dystrophin expression
Source: EMBO Mol Med. 2021 Mar 16;13(5):e13228. doi: 10.15252/emmm.202013228 (PMC8103086; doi:10.15252/emmm.202013228)
Supplement: Supplementary file 2 — Expanded View Figures PDF [file EMMM-13-e13228-s002.pdf]

Expanded View Figures

Figure EV1. The *Dup18-30* mouse model shows generalized dystrophic muscle pathology and locomotor dysfunctions.

A Heart, gastrocnemius, and diaphragm cross sections were analyzed for dystrophin localization via immunostaining in 15-week-old WT and *Dup18-30* mice. A representative sample is shown. Scale bar, 100 μm.

B The muscle morphology of the gastrocnemious and diaphragm was further investigated via H&E. A representative image is shown. Scale bars, 50 μm.

C, D The mice were tested in an open-field chamber in which total resting time (C) and average speed (D) were assessed. WT, n = 9; *Dup18-30*, n = 12. Data are represented as means ± SD. Statistical analyses were performed with Student's t-test. $^{**}P < 0.01$, $^{***}P < 0.001$.

▶

© 2021 The Authors

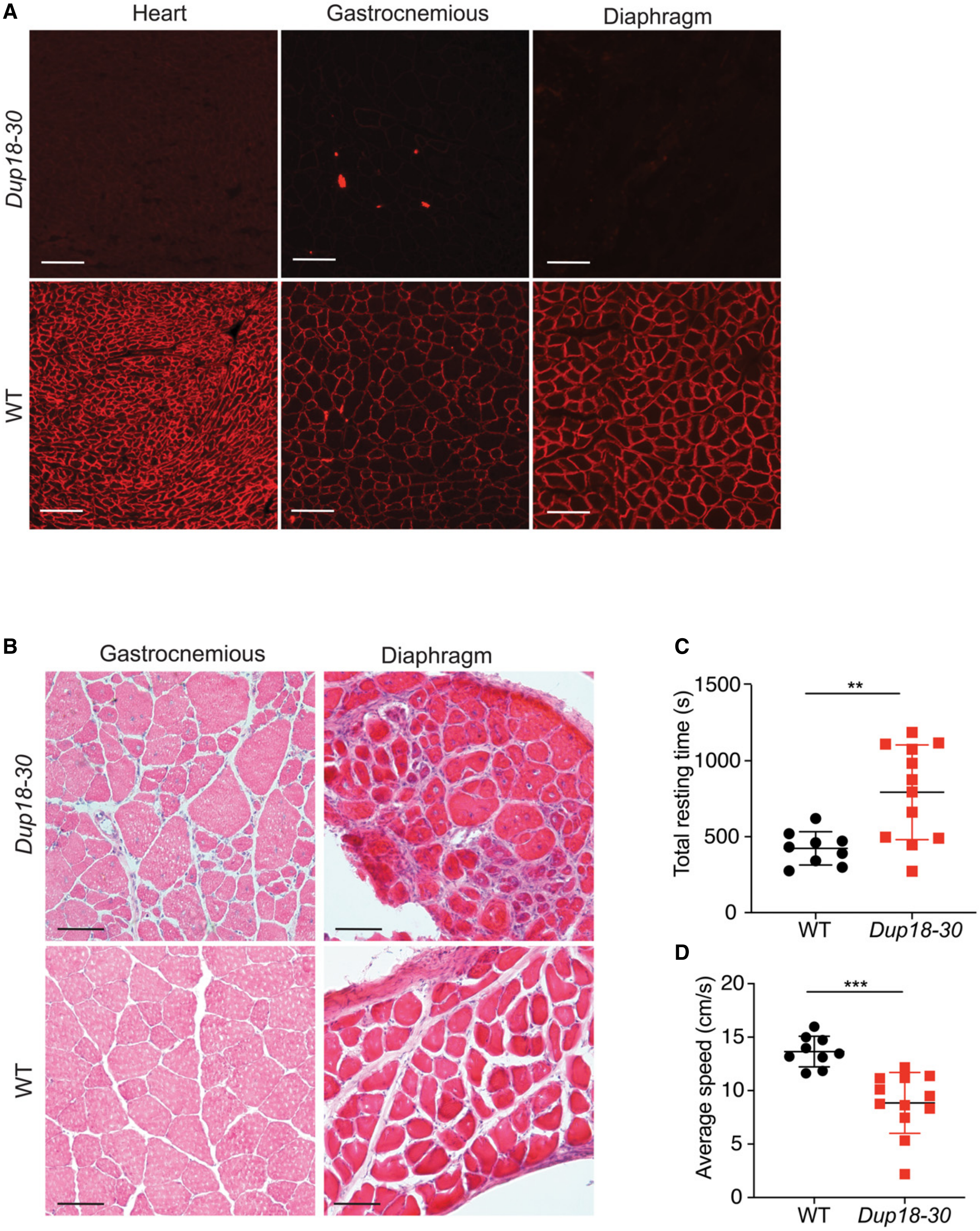
A
Heart
Gastrocnemious
Diaphragm
Dup18-30
WT
B
Gastrocnemious
Diaphragm
Dup18-30
WT
C
Total resting time (s)
1500
1000
500
0
**
WT
Dup18-30
D
Average speed (cm/s)
20
15
10
5
0
***
WT
Dup18-30

Figure EV1.

Figure EV2. The single-sgRNA treatment restores dystrophin expression in the triceps and diaphragm of *Dup18-30* mice.

A, B Western blotting detected restoration of dystrophin expression in (A) triceps and (B) diaphragm in the *Dup18-30* mice. 25% and 50% of the WT proteins compared to *Dup18-30* mice have been loaded on the gel. Calnexin was used as a loading control.

C Quantification of dystrophin Western blot (A and B). *Dup18-30* untreated, $n = 2$; *Dup18-30* treated, $n = 4$.

D Immunostaining showed restoration of dystrophin expression in the triceps and diaphragm. A representative image is shown. Scale bars, 100 μm.

E Percentage of dystrophin-positive fibers from the immunostaining in the triceps and diaphragm of *Dup18-30*-untreated and *Dup18-30*-treated mice. *Dup18-30* untreated, $n = 4$–5; *Dup18-30* treated, $n = 6$–10.

Data information: All data are represented as the mean ± SD. Statistical analyses were performed with Student's *t*-test. $^*P < 0.05$, $^{***}P < 0.001$.

© 2021 The Authors

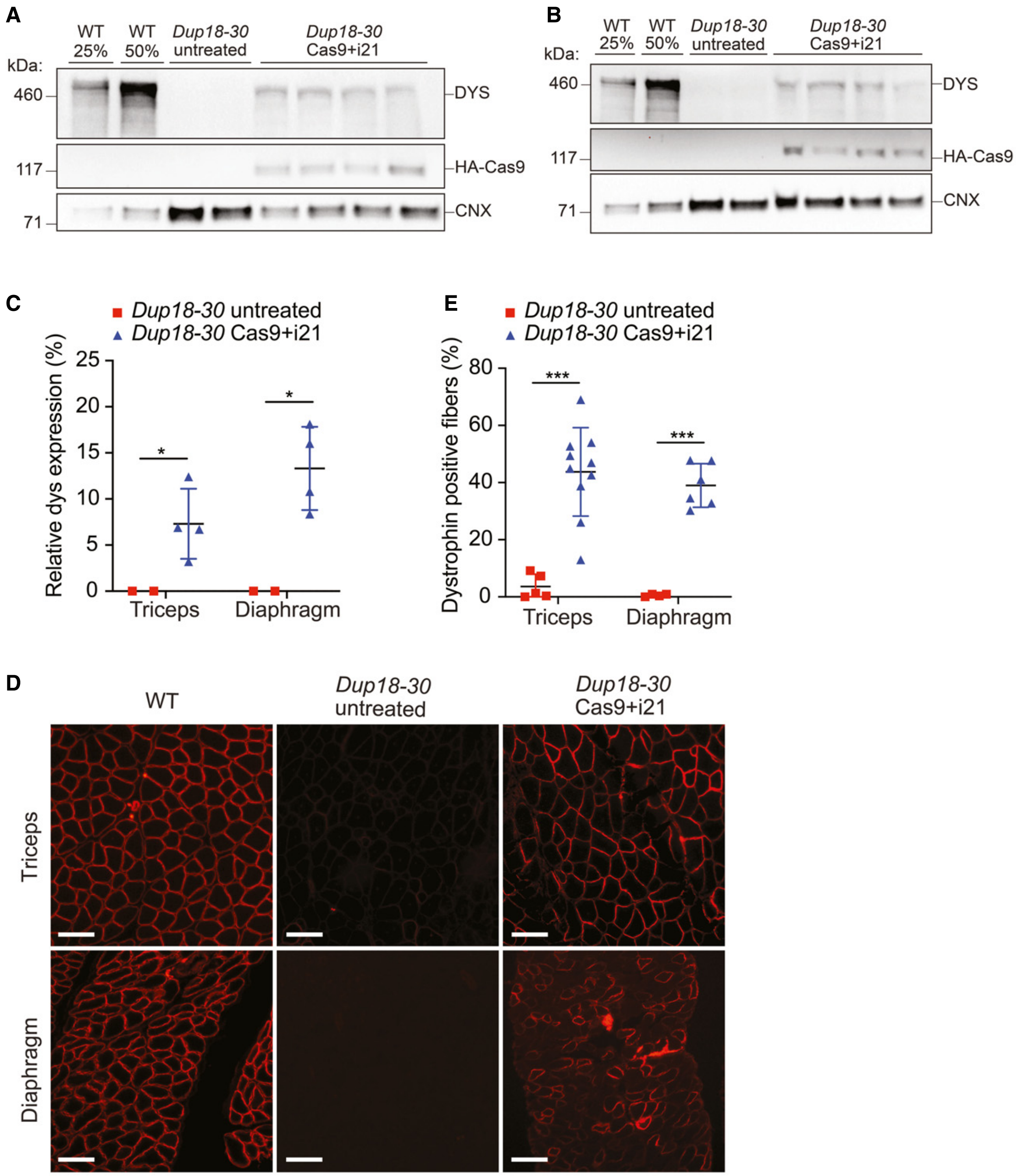
A
WT 25%
WT 50%
Dup18-30 untreated
Dup18-30 Cas9+i21
kDa:
460
117
71
DYS
HA-Cas9
CNX
B
WT 25%
WT 50%
Dup18-30 untreated
Dup18-30 Cas9+i21
kDa:
460
117
71
DYS
HA-Cas9
CNX
C
Dup18-30 untreated
Dup18-30 Cas9+i21
Relative dys expression (%)
25
20
15
10
5
0
Triceps
Diaphragm
E
Dup18-30 untreated
Dup18-30 Cas9+i21
Dystrophin positive fibers (%)
80
60
40
20
0
Triceps
Diaphragm
D
WT
Dup18-30 untreated
Dup18-30 Cas9+i21
Triceps
Diaphragm

Figure EV2.

Figure EV3. The Cas9 treatment restores expression of DGC components.

A Immunofluorescence staining for alpha-syntrophin and beta-sarcoglycan in the diaphragm and heart of WT, *Dup18-30*-untreated, and *Dup18-30*-treated mice. A representative image is shown. Scale bars, 100 μm.

B Immunofluorescence staining for nNOS in TA, triceps, and diaphragm of WT, *Dup18-30*-untreated, and *Dup18-30*-treated mice. A representative image is shown. Scale bars, 100 μm.

 © 2021 The Authors

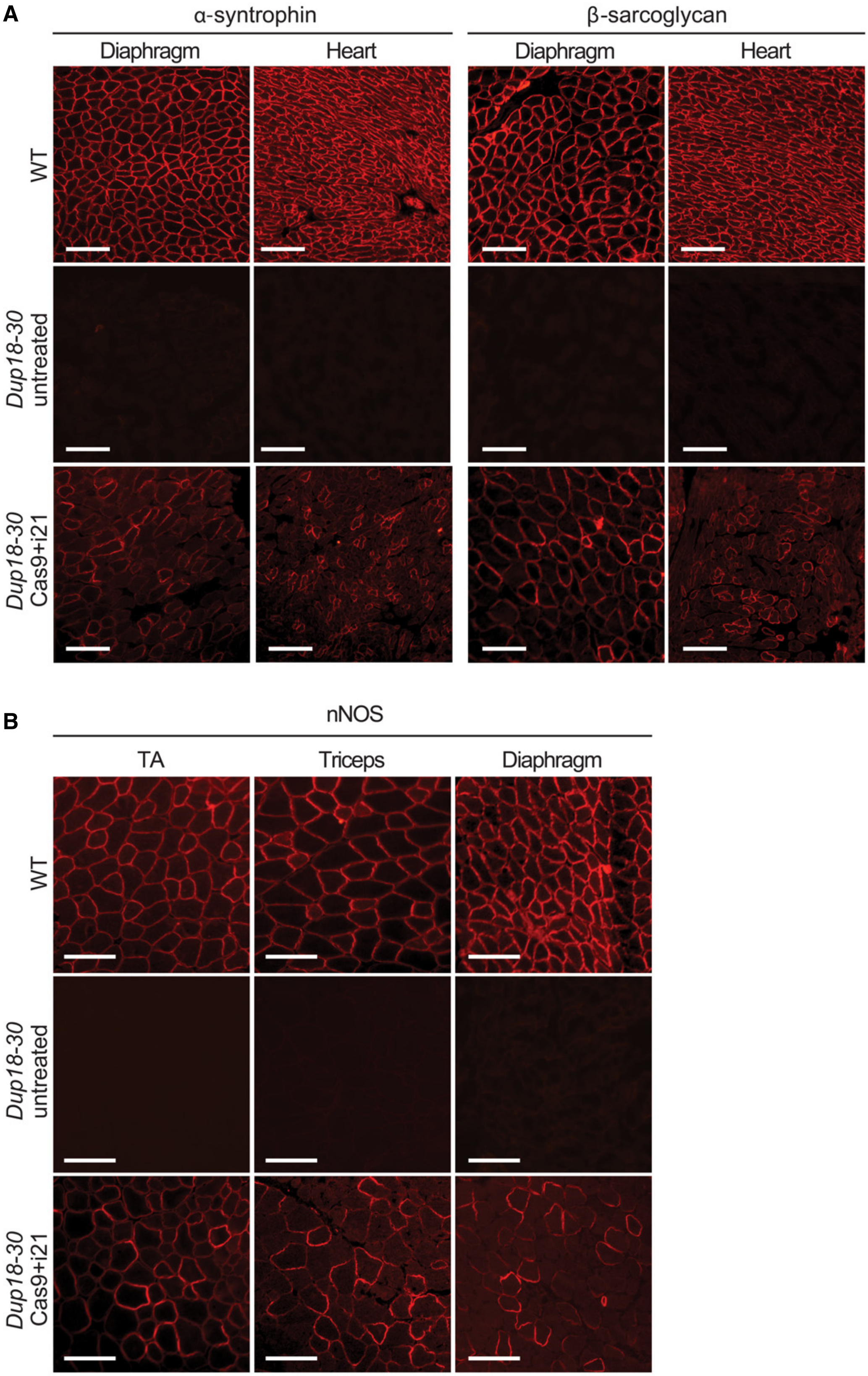
A
α-syntrophin
β-sarcoglycan
Diaphragm
Heart
Diaphragm
Heart
WT
Dup18-30 untreated
Dup18-30 Cas9+i21
B
nNOS
TA
Triceps
Diaphragm
WT
Dup18-30 untreated
Dup18-30 Cas9+i21

Figure EV3.

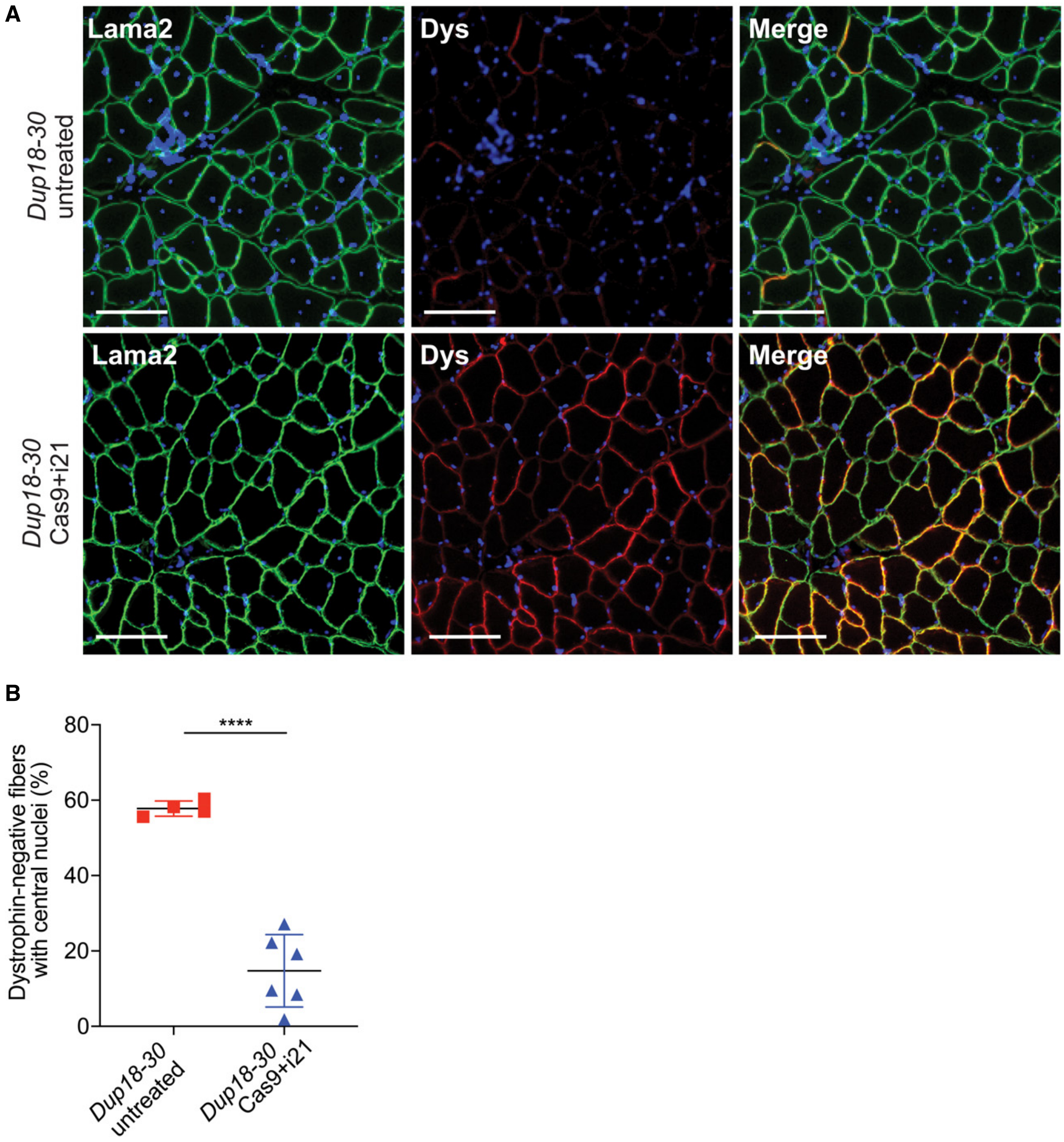

Figure EV4. The Cas9 treatment reduces central nuclei in dystrophin-negative muscle fibers.

A Immunofluorescence staining for laminin alpha-2 and dystrophin in the TA of *Dup18-30*-untreated, and *Dup18-30*-treated mice. A representative image is shown. Scale bars, 100 μm.

B Quantification of central nuclei in dystrophin-negative fibers in *Dup18-30*-untreated and *Dup18-30*-treated mice. *Dup18-30* untreated, $n = 5$; *Dup18-30* Cas9 treated, $n = 6$. All data are represented as the mean ± SD. Statistical analyses were performed with Student's *t*-test. ****$P < 0.0001$.

 © 2021 The Authors

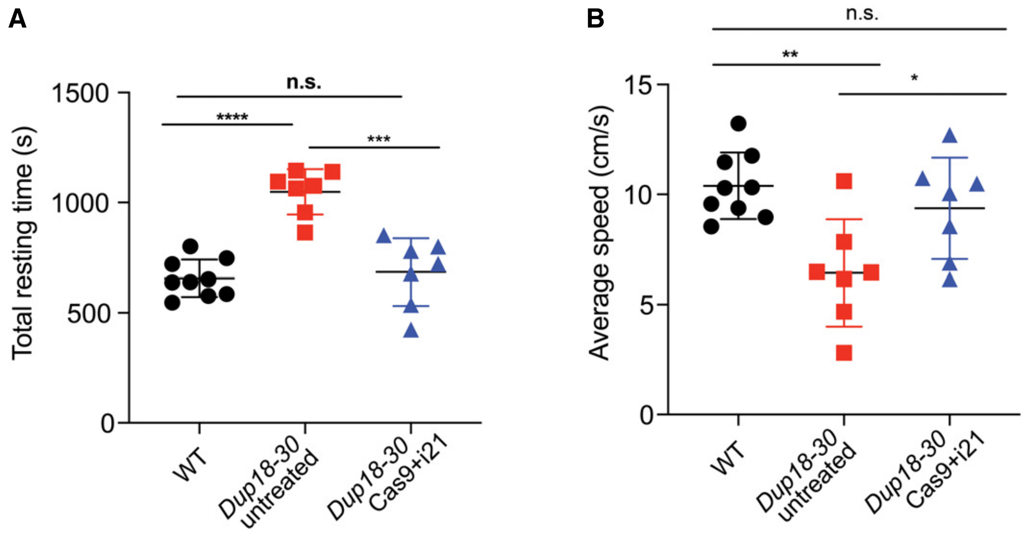

Figure EV5. The Cas9 treatment improves DMD disease phenotypes.

A, B WT, *Dup18-30*-untreated, and *Dup18-30*-treated mice were tested 7 weeks post-treatment in an open-field chamber measuring (A) total resting time in the arena and (B) average speed. WT, $n = 9$; *Dup18-30* untreated, $n = 7$; *Dup18-30* Cas9 treated, $n = 7$. All data are represented as the mean ± SD. Statistical analyses were performed with Student's *t*-test. n.s. not significant, $*P < 0.05$, $**P < 0.01$, $***P < 0.001$, $****P < 0.0001$.
